# Identification and Analysis of p53-Regulated Enhancers in Hepatic Carcinoma

**DOI:** 10.3389/fbioe.2020.00668

**Published:** 2020-06-30

**Authors:** Yin Zhang, Mingming Qian, Fei Tang, Qingqing Huang, Wenzhu Wang, Yanjing Li, Zhixue Li, Beiping Li, Zhengliang Qiu, Junjie Yue, Zhiyun Guo

**Affiliations:** ^1^School of Life Sciences and Engineering, Southwest Jiaotong University, Chengdu, China; ^2^Beijing Institute of Biotechnology, Beijing, China; ^3^Laboratory Animal Center, Academy of Military Medical Sciences, Beijing, China; ^4^Xinxiang Key Laboratory of Pathogenic Microbiology, Xinxiang, China

**Keywords:** p53, enhancer, hepatic carcinoma, transcription factors, microRNA

## Abstract

Enhancers can act as cis-regulatory elements to control transcriptional regulation by recruiting transcription factors (TFs) in a distance and orientation-independent manner. However, it is still unclear how p53 participates in the enhancer network as TF in hepatic carcinoma under the condition of DNA damage. A total of 14,286 active enhancers were identified through the integration of stable and unstable enhancer RNAs (eRNAs) captured by CAGE and GRO-seq, respectively. Furthermore, 218 p53-bound enhancers (Enh_p53_) were identified by analyzing p53 ChIP-seq in HepG2 cells after DNA damage. The results showed that the enhancer expression and histone markers of enhancers (H3K4me1, H3K4me2, H3K4me3, H3K9ac, and H3K27ac) revealed significantly higher level on Enh_p53_ than Enh_no−p53_ which suggested that p53 participated in regulating enhancer activity and chromatin structure. By analyzing 124 TFs ChIP-seq from ENCODE, 93 TFs were found significantly enriched on Enh_p53_ such as GATA4, YY1, and CTCF, indicating p53 may co-regulate enhancers with TFs participation. Moreover, significantly differentially expressed 438 miRNAs and 1,264 mRNAs were identified by analyzing small RNA-seq and RNA-seq, and 26 Enh_p53_-miRNAs and 145 Enh_p53_-mRNA interactions were identified by the integration of 3D genome data and genomic distance. The functional enrichment analysis showed that these miRNA targets and mRNAs were significantly involved in tumor biological processes and signaling pathways such as DNA replication, p53 signaling pathway, hepatitis B, focal adhesion, etc. The above results indicated that p53 participated in regulating enhancer network in hepatic carcinoma and Enh_p53_ exhibited significantly different characteristics with Enh_no−p53_.

## Introduction

Enhancers can act as tissue-specific cis-regulatory elements to positively regulate gene expression by recruiting TFs and their cofactors in a distance and orientation-independent manner. Previous studies have shown that most active enhancers can transcribe RNA, namely enhancer RNA (eRNA) under the mediation of transcription factor (TF) (Wang et al., 2011). Studies have shown that the expression of eRNA which is related to the activity of enhancer and dysregulation of the expression of enhancer can lead to the occurrence of various cancers including hepatic carcinoma (Kim et al., 2010).

The structural integrity and stability of DNA are critical for cell survival and physiological functions. DNA can be damaged under various stresses to trigger DNA repair which aims to maintain the integrity of cellular function and immune response (Bartek and Lukas, 2007). The p53 tumor suppressor, as sequence-specific DNA-binding TF, plays a key role in the entire DNA damage repair process (Giono and Manfredi, 2006). A previous study found that most p53-DNA binding sites have consistent signal characteristics with enhancer regions and confirmed that these p53-bound regions had enhancer activity (Melo et al., 2013). Recently, follow-up studies confirmed that a large number of p53 participated in regulating enhancer activity by binding to enhancer regions under doxorubicin-induced DNA damage, suggesting DNA damage may induce p53, which is then involved in enhancer regulatory network (Younger and Rinn, 2017). However, it is still unclear the characteristics and mechanisms of p53-enhancer regulatory network in hepatic carcinoma under the condition of DNA damage.

In this study, we identified a series of activity enhancers through the identification of stable and unstable eRNAs based on cap analysis gene expression (CAGE) and global run-on sequencing (GRO-seq) data in hepatic carcinoma, and two categories were divided according to whether the enhancer bound to p53 or not (Enh_p53_ and Enh_no−p53_). The results showed that the enhancer expression and histone markers of enhancers revealed significantly higher level on Enh_p53_ than Enh_no−p53_. TF enrichment analysis showed that p53 regulated enhancer activity and chromosome accessibility by directly or indirectly interacting with various TFs and co-factors. Finally, a series of differentially expressed microRNAs (miRNAs) and mRNAs regulated by Enh_p53_ were identified and the results indicated that they were related to tumorigenesis and the development of hepatic carcinoma significantly.

## Materials and Methods

### Data Sources

Raw global run-on sequencing (GRO-seq) and p53 ChIP-seq SRA files were downloaded from GEO data set (GSM2428726 and GSE64877). Histone modification, TFs ChIP-Seq and raw Hi-C data (ENCFF419ZIV and ENCFF122SLQ) in HepG2 cells were downloaded from the ENCODE project (GRCh38). Raw miRNA-seq and RNA-seq data under conditions of DNA damage were obtained from our previous study (Yang et al., 2016).

### Identification of Active Enhancers in HepG2

Enhancers with stable and unstable transcripts were identified based on extracting CAGE and GRO-seq data. Enhancers that were identified by CAGE method were obtained from the HACER database *(**http://bioinfo.vanderbilt.edu/AE/HACER/**)* (Wang et al., 2019). GRO-seq SRA files that were downloaded from the GEO data set were converted into FASTQ format by using sratoolkit (Leinonen et al., 2010). After adapter trimming and reads with a quality score below 10 being removed by Cutadapt (Martin, 2011), reads that longer than 15bp were aligned to the human genome (hg38) by using Bowie2 (Langmead et al., 2009). Reads with mapping quality <10 were removed, and enhancers were identified by using NRSA *(**http://bioinfo.vanderbilt.edu/NRSA/**)* (Wang et al., 2018).

### Histone Modification, TF and Motif Analysis of Enhancer

p53-bound enhancers were identified when there appeared the intersection of p53 binding sites and the enhancer regions. Histone modification, TF binding were obtained by calculating the signal within 1 kb upstream and downstream of the enhancer. Enriched TF motifs in enhancer were identified by using AME (Bailey et al., 2009) based on known TF motifs which were obtained from the HOCOMOCO database (Kulakovskiy et al., 2016).

### Differential Expression Analysis of miRNA and mRNA

Raw sequence reads with low quality were filtered by FastQC (Andrews, 2010). Adapter sequences and low quality reads were removed by using Cutadapt. The trimmed miRNA-seq and RNA-seq reads were mapped to the reference genome by using Bowtie (Langmead, 2010) and HISAT2 (Kim et al., 2015), respectively. The number of reads that were mapped to each gene was counted by using HTSeq (Anders et al., 2015). Differential expression analysis was performed by using edgeR (q-value <0.05 and log2|fold change| ≥ 1).

### Hi-C Data Processing and TAD Identification

Raw paired reads were downloaded from ENCODE, then Python code was used to pre-truncate the reads that contain potential ligation junctions. The read pairs were mapped to reference genome using Bowtie2 (Langmead and Salzberg, 2012). Unmapped and low-quality mapped reads with map quality <30 were filtered out and the paired reads are selected using SAMtools (Li et al., 2009) and Unix code. Data normalization, visualization, and TAD analysis were performed by HiCtool (Calandrelli et al., 2018).

### Identification of Differentially Expressed miRNAs and mRNAs Regulated by Enhancer

Three methods were used to identify enhancer-regulated miRNAs in our study. FANTOM miRNA transcriptional start site (TSS) annotation was used and miRNA promoters were defined as 1 kb upstream and 500 bp downstream of the TSS of a miRNA. (1) If the enhancer and miRNA promoter were located within the same TAD region, then miRNAs would be considered to be regulated by enhancers. (2) Enhancer-regulated miRNAs were identified if enhancer and miRNA promoter were located in interaction regions based on the 4DGenome (Teng et al., 2015), a general repository for chromatin interaction data. (3) Enhancer-miRNA regulation was identified based on a previous study which provided the linkage score for enhancer-miRNA interaction as the following formula in below, where A and B were the distance from the enhancer center to the closest miRNA gene and the closest gene, respectively. According to the research of Suzuki (Suzuki et al., 2017), miRNA genes with S value below 0.2 were categorized as enhancer-associated miRNAs.

$$\begin{array}{l} \;S\;\left(b÷a\right)\;=\;\left(B-A\right)÷\;\left(A+B\right) \end{array}$$

Enhancer-regulated mRNAs were identified according to the following methods, mRNA TSS annotation was obtained from GENCODE database and promoters of mRNA were defined as 1 kb upstream and 500 bp downstream of the TSS of a gene. (1) If the enhancer and mRNA promoter were located within the same TAD region, that mRNAs were considered to be regulated by enhancers. (2) Enhancer-regulated mRNAs were identified if enhancer and mRNA promoter were located in interaction regions based on the 4DGenome. (3) If a mRNA promoter was located within 100 kb upstream or downstream of an enhancer, this mRNA was considered to be regulated by the enhancer (Chepelev et al., 2012). The final enhancer-regulated miRNAs and mRNAs is the union of the results of three methods.

## Results and Discussion

### Identification of Active Enhancer in HepG2 in DNA Damage

**Figure 1:**
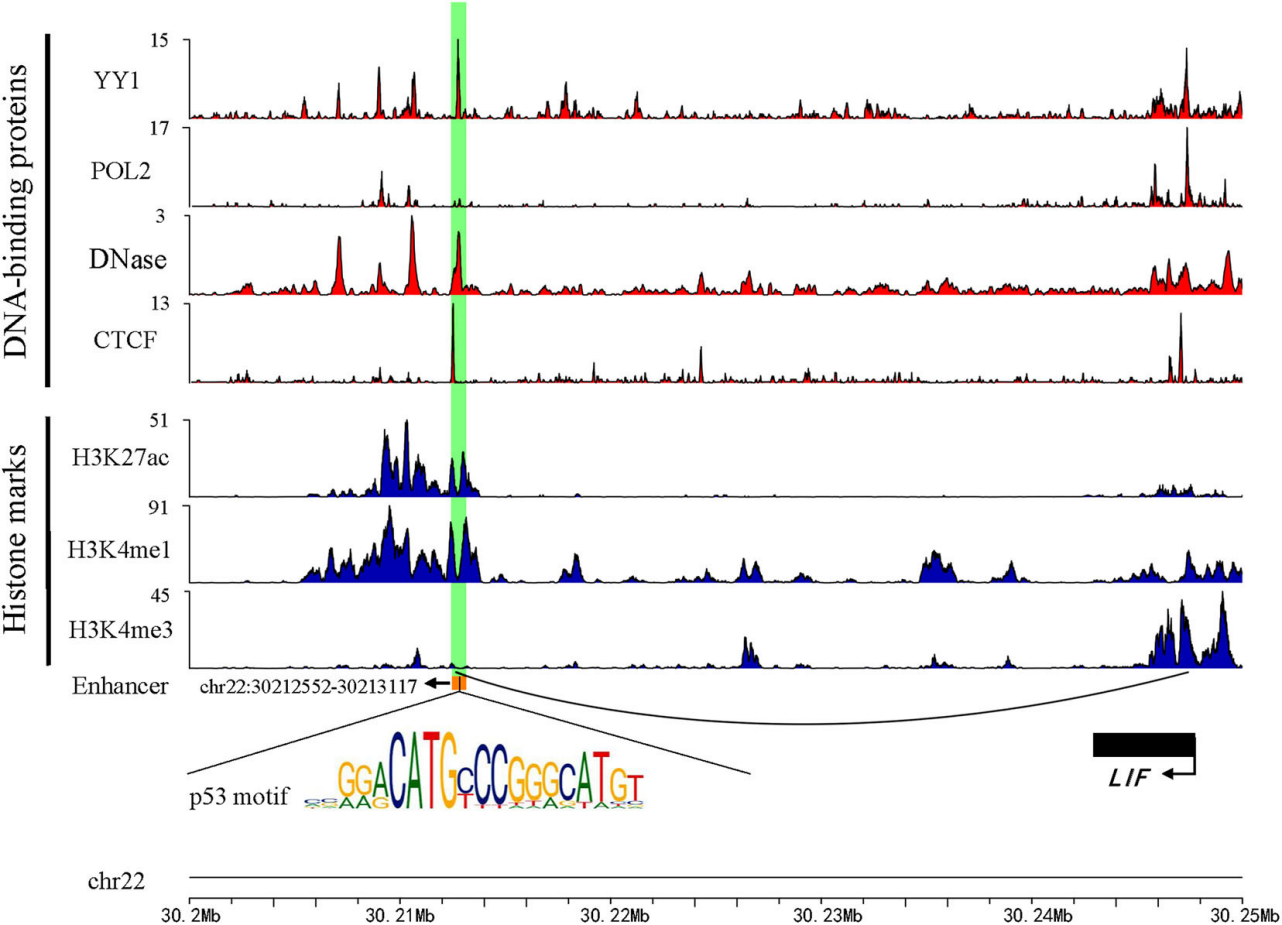
An example genomic map of enhancer chr22: 30212552-30213117. Blue represents the histone modification, red marks the TF binding on the chromosome, and the black arc represents a Topology domain.

Previous studies have shown that active enhancers produce both stable and unstable transcripts. CAGE is suitable for capturing stable transcripts, while GRO-seq is more suitable for capturing unstable transcripts (Li et al., 2016). Based on these studies, more comprehensive active enhancers were identified by integrating data obtained from these two experimental methods. Firstly, 13,088 active enhancers identified by CAGE in HepG2 were download from the HACER database. Next, 1,321 active enhancers from GRO-seq were identified by obtaining raw data from GEO and NRSA, which were used to identify enhancers (Table S1). Finally, a total of 14,286 enhancers were identified through merging two types of enhancers and converted from hg19 to hg38 by using LiftOver (Table S2). As expected, the active enhancers we identified had known enhancer features, including DNase hypersensitive sites, high levels of H3K27ac, H3K4me1, low levels of H3K4me3, and enrichment of YY1 and CTCF that mediated long-range enhancer-promoter interactions (Figure 1).

### p53-bound Enhancer (Enh_p53_) Has Higher Transcriptional Activity Than Enh_no-p53_

**Figure 2:**
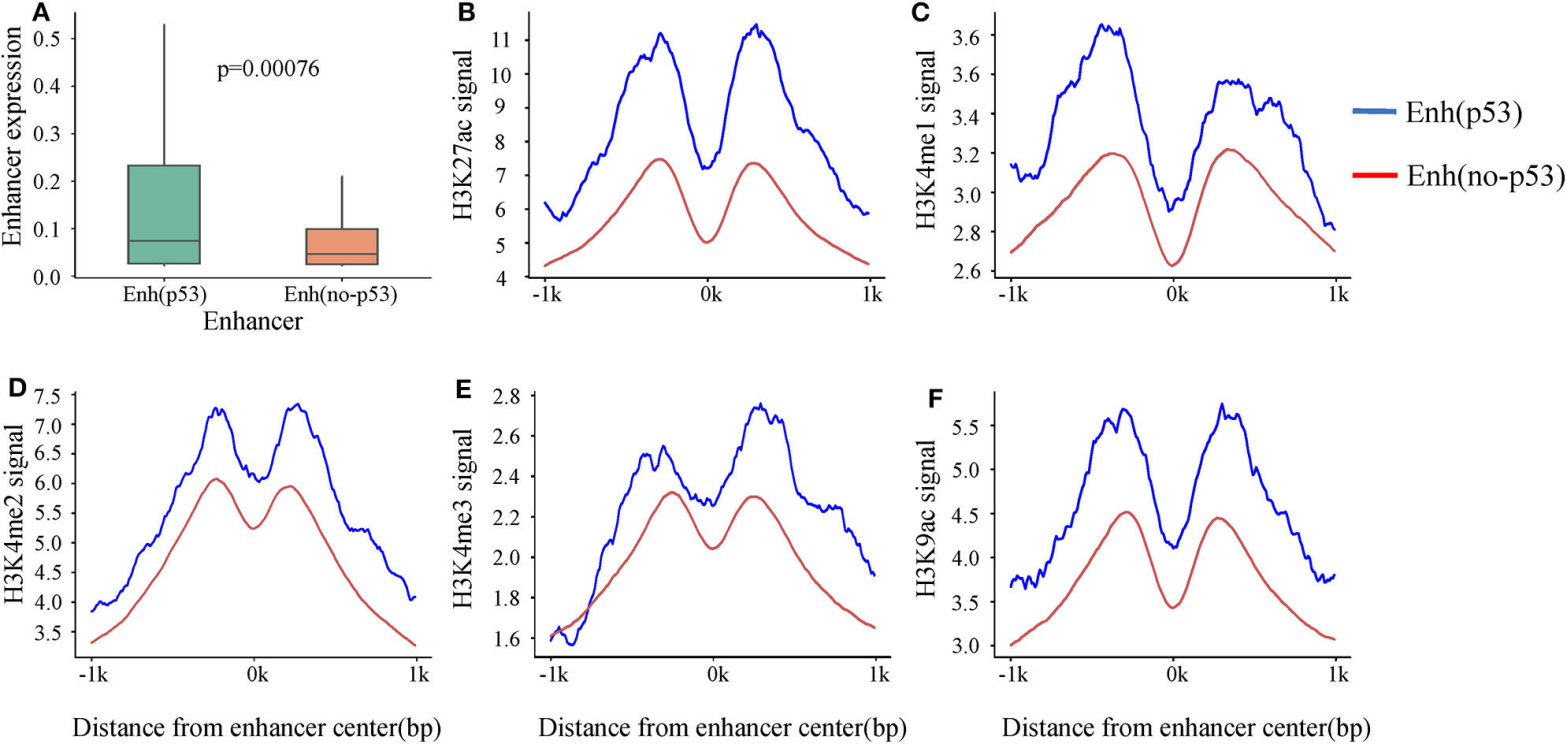
The differences in expression and histone modification of the two types of enhancers. **(A)** The expression of the two types of enhancers. *p*-value is calculated by Wilcoxon test. **(B–F)** Different histone modification on the two types of enhancers. The blue line represents Enh_p53_, and the red line marks Enh_no−p53_.

A recent study showed that p53 can bind to enhancers to regulate tumorigenesis and development (Younger and Rinn, 2017). To investigate the p53-bound enhancer in hepatic carcinoma under DNA damage, we matched 14,286 enhancers with 13,723 p53 binding sites obtained from HepG2 cell in the condition of DNA damage. Finally, 218 p53-bound enhancers (Enh_p53_) were identified (Table S3). To deeply study the effect of p53 on enhancer function, we classified enhancers into two categories based on whether they bound p53 or not: Enh_p53_ and Enh_no−p53_. Furthermore, expression levels of these two types of enhancers were compared, and the results showed that the expression level of Enh_p53_ was higher than Enh_no−p53_ significantly (Figure 2A), suggesting that the binding of p53 further promoted the enhancer activity.

Previous studies revealed that activity of enhancers are always associated with histone modifications (Creyghton et al., 2010). To investigate if p53 could cause changes of histone modifications surrounding enhancer sequence, we downloaded nine histone modification ChIP-seq data of HepG2 from ENCODE including H3K4me1, H3K4me2, H3K4me3, H3K9ac, H3K9me3, H3K27ac, H3K27me3, H3K36me3, and H4K20me1. It was found that five histone modifications (H3K4me1, H3K4me2, H3K4me3, H3K9ac, and H3K27ac) which had been proven to be markers of identification of active enhancers (Creyghton et al., 2010) showed significant signal peaks on enhancer, and all the signals of these five histone modifications were significantly stronger on Enh_p53_ than Enh_no−p53_ (Figures 2B–F). The above results suggested that p53 could affect enhancer activity and might be regulated through changing histone modifications surrounding enhancer sequence.

### Transcription-Factors Binding Analysis of p53-bound Enhancer

**Figure 3:**
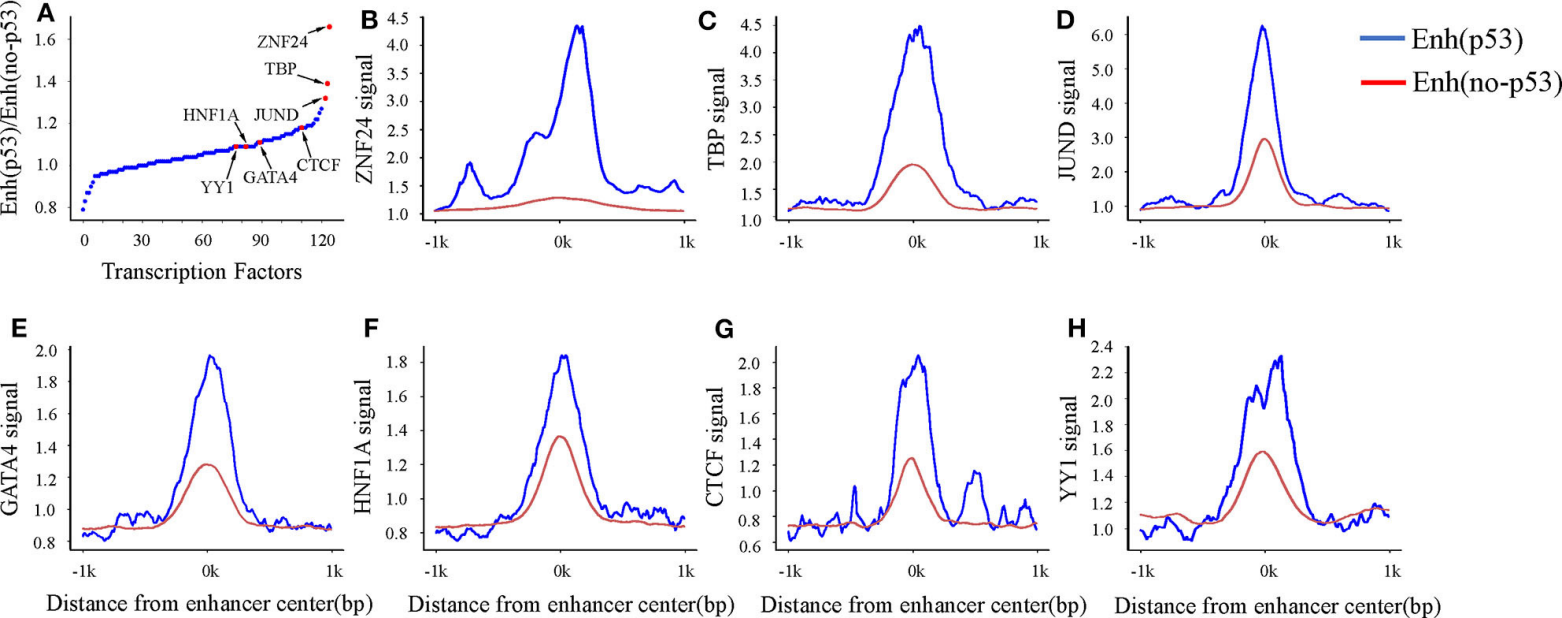
TF binding on Enh_p53_ and Enh_no−p53_. **(A)** Ratios of TF binding signals on the Enh_p53_ and Enh_no−p53_ by calculating the mean value of binding signals within 1 kb upstream and downstream of the enhancer. **(B–H)** TF binding signals located in the region of ± 1 kb from the enhancer center.

A previous study has shown that enhancers interact with the gene promoter by recruiting TFs to regulate gene expression (Nolis et al., 2009). To study the effect of TF binding on Enh_p53_/Enh_no−p53_ and find which TFs cooperate with p53 as a cofactor to regulate enhancer activity, we compared the TFBS on both types of enhancers. The result showed that 124 TFs could bind to the enhancers by analyzing TF ChIP-seq signals located in the region of ± 1kb from the enhancer center. 93 out of 124 TFs had higher binding signals on Enh_p53_ than Enh_no−p53_ (Figure 3A). The other 31 TFs had lower binding signals on Enh_p53_ than Enh_no−p53_. Notably, ZNF24, TBP, and JUND had the most significant signal ratio compared with other TFs (Figures 3B–D). Moreover, several TFs associated with enhancer functions, such as GATA4, HNF family, YY1 and CTCF, also presented stronger TF binding signals on Enh_p53_ compared with Enh_no−p53_ (Figures 3E–H).

Studies have shown that the GATA4 and HNF protein families could promote the formation of open chromatin (Rogerson et al., 2019). Another study showed that p53 could bind to “pioneering sites,” a chromosome inaccessible region in advance and p53 binding sites shifts from inaccessibility to accessibility in response to DNA damage (Younger and Rinn, 2017). In summary, p53 may be involved in the change of chromatin accessibility with the participation of GATA4, HNF4A, and HNF1A in response to DNA damage. Besides, several structural regulators of enhancer-promoter loops such as YY1 and CTCF also revealed higher signal on Enh_p53_ than Enh_no−p53_, suggesting p53 may play a key role in mediating long-range chromatin looping.

**Figure 4:**
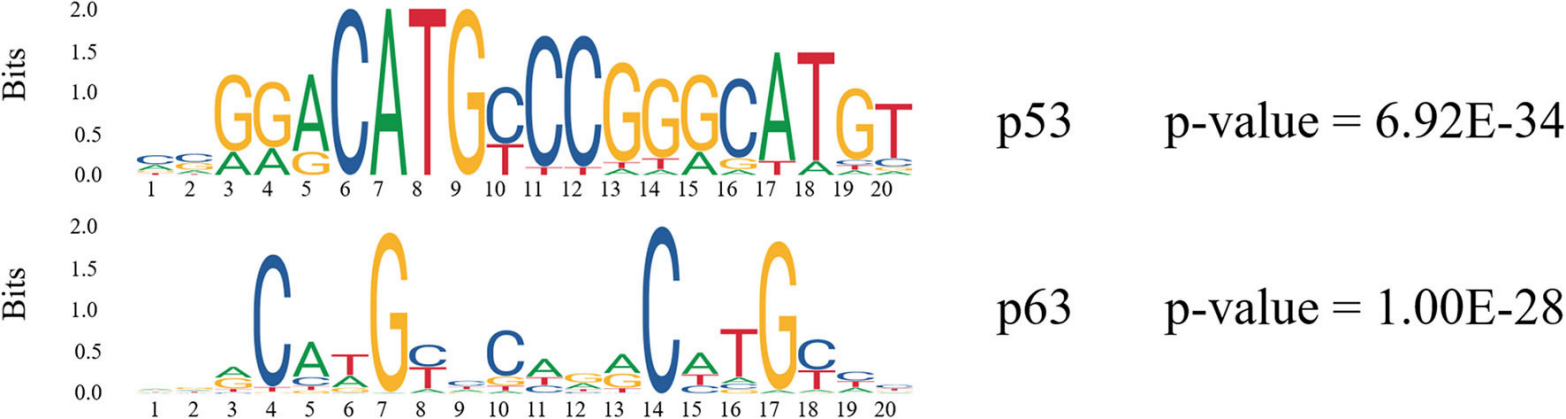
Motif analysis on p53-bound enhancer. The consensus logo represents the degree of conservation of each position using the height of the consensus character at that position.

In order to find more TFs binding to enhancers, we analyzed the TF motifs on both types of enhancers using MEME. Finally, motifs of 200 TFs were obtained on Enh_p53_ and motifs of 492 TFs were obtained on Enh_no−p53_ from HOCOMOCO database (Table S4). It was found that p53 and p63 only appeared on Enh_p53_ compared with other TFs which were present in both two types of enhancers (Figure 4). p63 is an indispensable factor in p53-dependent apoptosis in response to DNA damage (Meek, 2004), suggesting that p53 performs regulation on enhancers with p63's assistance. In summary, p53 may cooperate with other TFs to participate in the regulation of enhancers and chromosome looping.

### Identification of Differentially Expressed miRNAs and mRNAs Regulated by Enh_p53_

Recent studies have found that enhancers (typical enhancers and super enhancers) regulate miRNA expression and participate in the synthesis of miRNAs regulated by Drosha / DGCR8 (Suzuki et al., 2017). Notably, previous study had proven that p53 also driver the Drosha/DGCR8-mediated primary miRNA processing like enhancer (Hermeking, 2007). These studies indicated that p53, enhancers, miRNAs, and mRNAs may form a complex network and play a critical role in tumorigenesis. To investigate this, we reanalyzed the miRNA-seq and RNA-seq data from previous studies in our laboratory in response to DNA damage (p53 activation) in HepG2 cells. Finally, significantly differentially expressed 438 miRNAs and 1,264 mRNAs were identified after activating p53 in HepG2. Among them, 76 miRNAs were significantly down-regulated and 362 miRNAs were significantly up-regulated. Meanwhile, 724 mRNAs were significantly up-regulated and 540 mRNAs were significantly down-regulated.

Furthermore, in order to identify the miRNA regulated by Enh_p53_ as comprehensively as possible, we identified the miRNA regulated by the enhancer through two regulation modes: distal and proximal enhancer-miRNA regulation. Finally, 26 Enh_p53_-miRNAs interactions were identified by the integration of three approaches (TAD, 4DGenome, and proximal distance, details see methods) (Table 1). Several miRNAs have shown to be post-transcriptionally upregulated in a p53-dependent and p68/p72-dependent manner with DNA damage such as miR-16-5p (upregulated 8.63-fold), miR-143-3p (upregulated 8.46-fold), and overexpression of these p53-induced miRNAs caused a decreasing rate of cell proliferation (Wan et al., 2011).

**Table 1:** 26 Enh_p53_-regulated miRNA identified by the integration of three approaches.

**Enhancer**			**miRNA**		**Approach**		
****Chr.****	**Start**	**End**	****miRNA name****	****log2 (FoldChange)****	**Distance**	**TAD**	**4DGemone**
chr1	161612422	161612716	hsa−miR−4654	2.20		√	
chr1	161612422	161612716	hsa−miR−556−5p	3.95		√	
chr2	239632126	239646548	hsa−miR−2467−5p	9.73	√	√	
chr2	239632126	239646548	hsa−miR−4786−5p	7.43		√	
chr7	128236919	128237387	hsa−miR−129−5p	3.77		√	
chr7	130373756	130374065	hsa−miR−335−3p	7.71		√	
chr7	130373756	130374065	hsa−miR−29a−3p	−4.01			√
chr7	130373756	130374065	hsa−miR−29b−3p	−4.02			√
chr7	130896838	130897634	hsa−miR−29a−3p	−4.01		√	
chr7	130896838	130897634	hsa−miR−29b−3p	−4.02		√	
chr7	135300381	135315387	hsa−miR−6509−5p	5.11	√	√	
chr8	95220062	95224485	hsa−miR−3150a−3p	3.75		√	
chr8	95220062	95224485	hsa−miR−3150a−5p	5.29		√	
chr11	66970815	66971421	hsa−miR−3163	3.75		√	√
chr11	118912202	118912669	hsa−miR−6716−3p	6.10		√	
chr13	50529144	50529602	hsa−miR−16−5p	3.12	√	√	
chr13	50529144	50529602	hsa−miR−3613−5p	5.21	√	√	
chr14	95265396	95265951	hsa−miR−3173−5p	3.04		√	
chr14	104348582	104353696	hsa−miR−203a−3p	4.72	√	√	
chr16	57893719	57894288	hsa−miR−6772−3p	6.11		√	√
chr17	56915657	56916183	hsa−miR−3614−5p	5.22	√	√	
chr17	57187353	57253506	hsa−miR−3614−5p	5.22		√	
chr19	782330	783005	hsa−miR−4745−5p	4.65	√	√	√
chr20	62651854	62652518	hsa−miR−4758−3p	4.95		√	
chr22	37898835	37899016	hsa−miR−659−5p	4.14		√	
chr22	37898835	37899016	hsa−miR−6820−5p	3.70		√	

Similar to the identification of Enh_p53_-miRNA, 145 Enh_p53_-mRNA pairs of 121 differentially expressed mRNAs were identified by the integration of chromosome interaction and distance (See methods, Table S5). Notably, there were five enhancers that regulated both miRNA and mRNA, involving a total of 7 miRNAs and 11 mRNAs (Table S6), suggesting that these enhancers might play a key role in the p53 network.

To analyze the function and the potential pathway of miRNA target genes and mRNAs regulated by Enh_p53_, Gene Ontology (GO) and Kyoto Encyclopedia of Genes and Genomes (KEGG) pathway functional enrichment analysis were performed by using the miRTarBase database (Hsu et al., 2011) and the R package clusterProfiler (Yu et al., 2012). GO and KEGG pathway analysis revealed that these miRNA targets and mRNAs were significantly involved in tumor biological processes such as DNA replication, folding, and chromatin assembly (FDR <0.05). KEGG pathway enrichment analysis showed that miRNA target genes and mRNAs regulated by Enh_p53_ significantly enriched in p53 or hepatic carcinoma related pathways such as p53 signaling pathway, focal adhesion, mTOR signaling pathway and hepatitis B (FDR <0.05, Figure S1). In addition, in order to study the effect of Enh_p53_-regulated miRNAs target genes and mRNAs on hepatocarcinogenesis, we obtained the driver genes affecting hepatocarcinogenesis from the driverDB database (Liu et al., 2020). The results showed that 32% of the driver genes are miRNA target genes and mRNA regulated by Enh_p53_, such as TP53, AGO2, EGFR, FOXP1, etc. In summary, the miRNA and mRNA regulated by Enh_p53_ are critical for the occurrence of liver cancer.

## Conclusion

In this study, a total of 218 p53-bound enhancers were identified by analyzing CAGE and GRO-seq data. It was found that Enh_p53_ had higher transcriptional activity and ability to bind TFs, and Enh_p53_ was also significantly different from Enh_no−p53_ in TF motif recognition and histone modifications. To further identify miRNAs and mRNAs regulated by Enh_p53_, we integrated multiple methods to identify 26 Enh_p53_-miRNAs and 145 Enh_p53_-mRNAs based on distal and proximal enhancers regulation. The results revealed that these miRNA targets and mRNAs were significantly involved in tumor biological processes and signaling pathways. However, these findings still required further finer experimental verification to prove this network. In summary, the results above indicated that p53 was involved in the hepatic tumorigenesis and development by mediating enhancers under DNA damage and provided a theoretical method basis for exploring the regulation of TFs and enhancers in the future studies.

## Data Availability Statement

All datasets generated for this study are included in the article/Supplementary Material.

## Author Contributions

ZG and JY conceived and designed the experiments. QH and WW acquired the experiment data. YZ, MQ, and FT performed the study and carried out the data analysis. ZG, YL, and ZL wrote this manuscript. ZG, ZQ, and BL revised the manuscript. All authors have read and approved the final manuscript.

## Conflict of Interest

The authors declare that the research was conducted in the absence of any commercial or financial relationships that could be construed as a potential conflict of interest.
