# Supplementary material for: Identification and Analysis of p53-Regulated Enhancers in Hepatic Carcinoma
Source: Front Bioeng Biotechnol. 2020 Jun 30;8:668. doi: 10.3389/fbioe.2020.00668 (PMC7338759; doi:10.3389/fbioe.2020.00668)
Supplement: Supplementary file 6 [file Table_6.pdf]

Table S6. Enh_{p53} that regulates both miRNA and mRNA

Enhancer	miRNA	Gene
chr7:130373756-130374065	hsa-miR-335-3<br>hsa-miR-29a-3p<br>hsa-miR-29b-3p	KLF14
chr7:135300381-135315387	hsa-miR-6509-5p	AGBL3
chr11:118912202-11891266	hsa-miR-6716-3p	UPK2 、 ABCG4
chr16:57893719-57894288	hsa-miR-6772-3p	ADGRG1、ADGRG3、CFAP20
chr19:782330-783005	hsa-miR-4745-5p	MADCAM1、GZMM、CFD、ARID3A
