# Supplementary material for: Identification and Analysis of p53-Regulated Enhancers in Hepatic Carcinoma
Source: Front Bioeng Biotechnol. 2020 Jun 30;8:668. doi: 10.3389/fbioe.2020.00668 (PMC7338759; doi:10.3389/fbioe.2020.00668)
Supplement: Supplementary file 7 [file Data_Sheet_1.PDF]

A

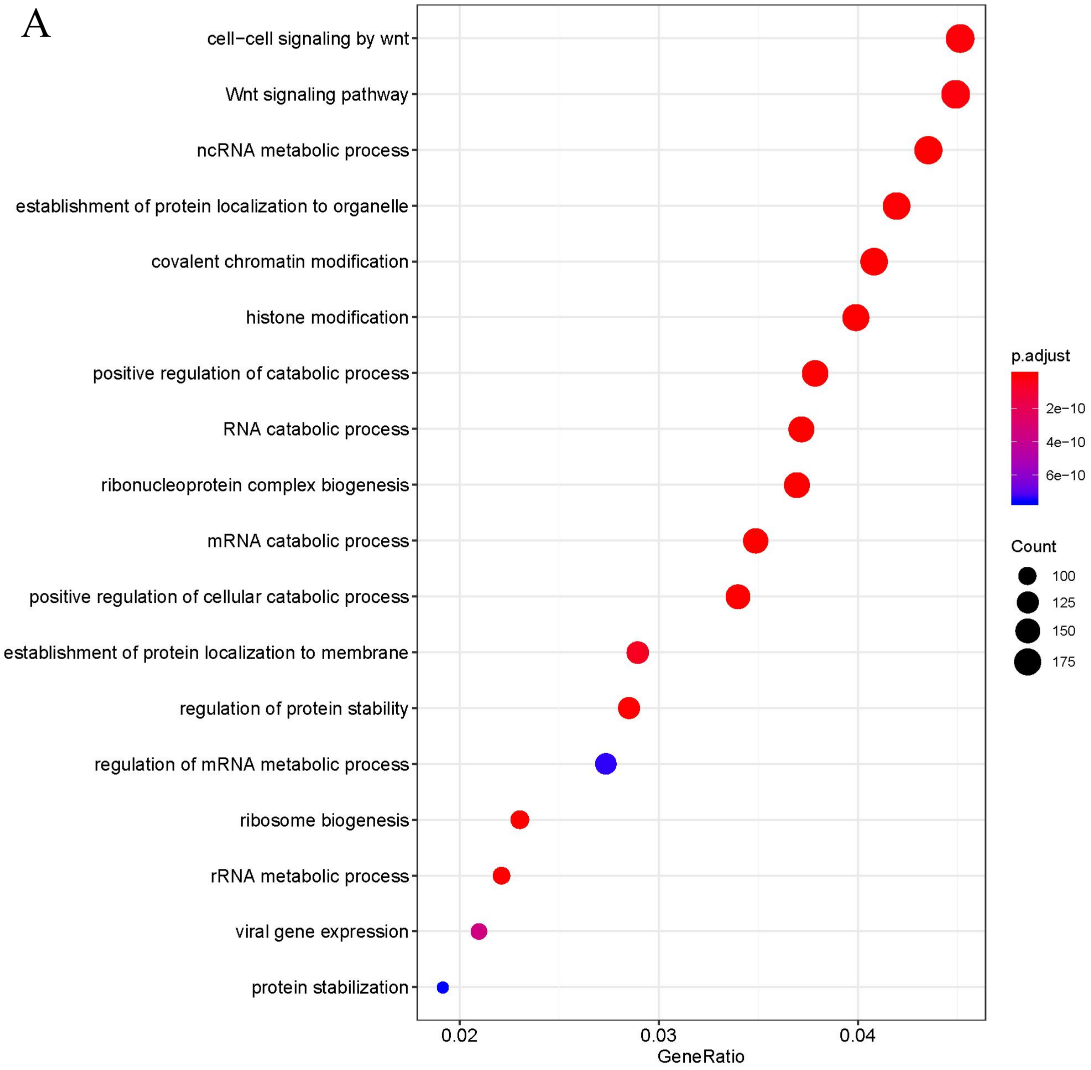
cell−cell signaling by wnt
Wnt signaling pathway
ncRNA metabolic process
establishment of protein localization to organelle
covalent chromatin modification
histone modification
positive regulation of catabolic process
RNA catabolic process
ribonucleoprotein complex biogenesis
mRNA catabolic process
positive regulation of cellular catabolic process
establishment of protein localization to membrane
regulation of protein stability
regulation of mRNA metabolic process
ribosome biogenesis
rRNA metabolic process
viral gene expression
protein stabilization
0.02
0.03
0.04
GeneRatio
p.adjust
2e−10
4e−10
6e−10
Count
100
125
150
175

B

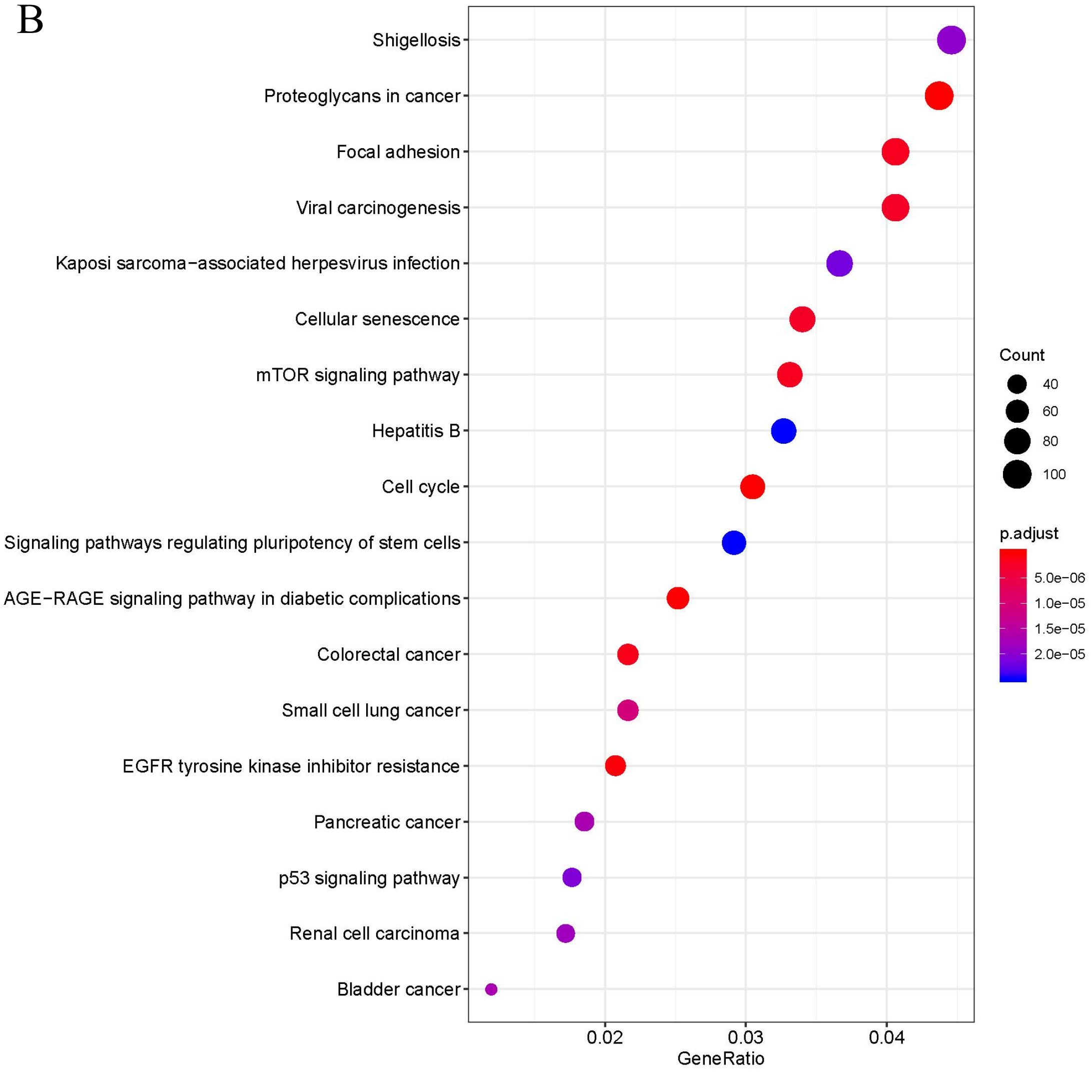
Shigellosis
Proteoglycans in cancer
Focal adhesion
Viral carcinogenesis
Kaposi sarcoma−associated herpesvirus infection
Cellular senescence
mTOR signaling pathway
Hepatitis B
Cell cycle
Signaling pathways regulating pluripotency of stem cells
AGE−RAGE signaling pathway in diabetic complications
Colorectal cancer
Small cell lung cancer
EGFR tyrosine kinase inhibitor resistance
Pancreatic cancer
p53 signaling pathway
Renal cell carcinoma
Bladder cancer
0.02
0.03
0.04
GeneRatio
Count
40
60
80
100
p.adjust
5.0e−06
1.0e−05
1.5e−05
2.0e−05

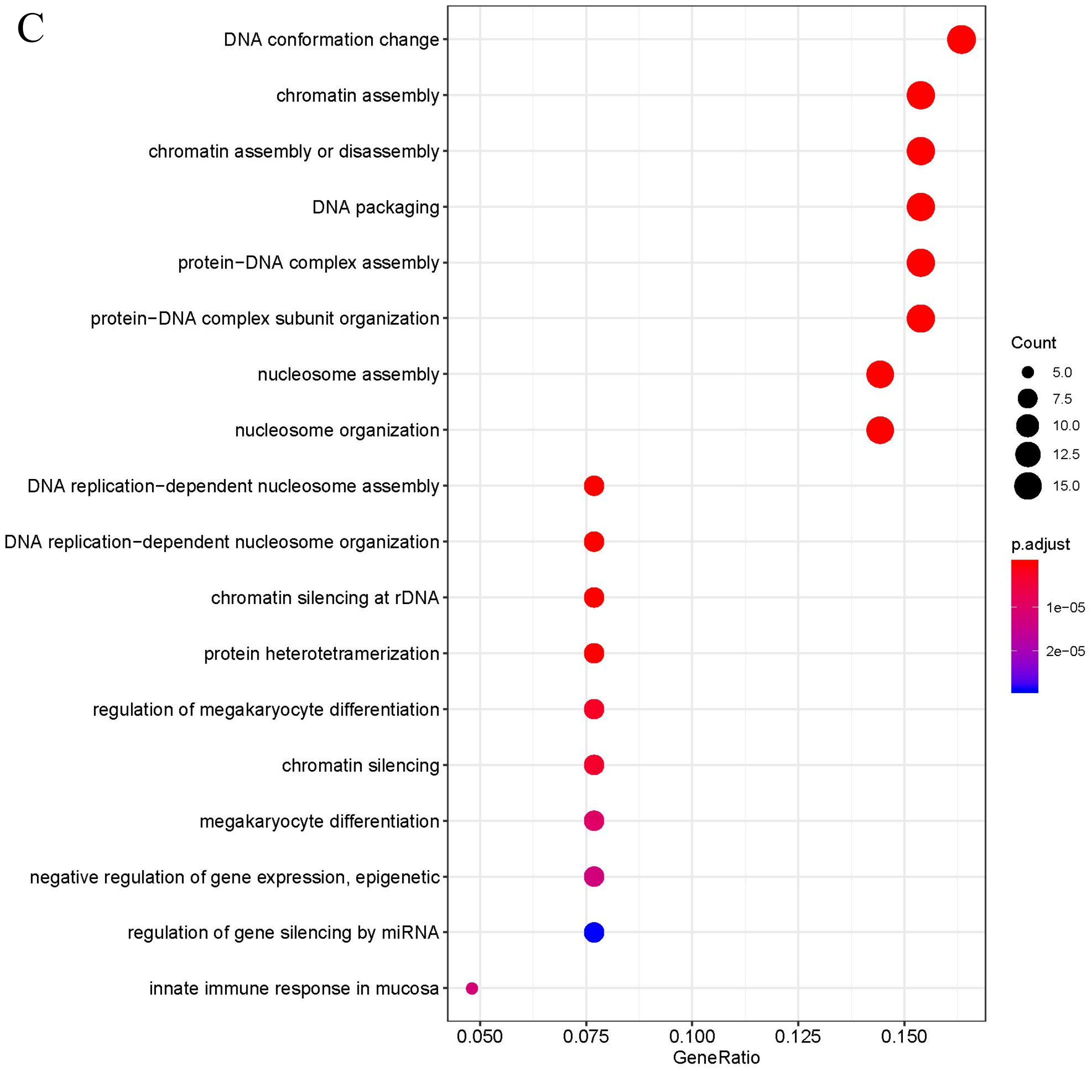
C
DNA conformation change
chromatin assembly
chromatin assembly or disassembly
DNA packaging
protein−DNA complex assembly
protein−DNA complex subunit organization
nucleosome assembly
nucleosome organization
DNA replication−dependent nucleosome assembly
DNA replication−dependent nucleosome organization
chromatin silencing at rDNA
protein heterotetramerization
regulation of megakaryocyte differentiation
chromatin silencing
megakaryocyte differentiation
negative regulation of gene expression, epigenetic
regulation of gene silencing by miRNA
innate immune response in mucosa
0.050
0.075
0.100
0.125
0.150
GeneRatio
Count
5.0
7.5
10.0
12.5
15.0
p.adjust
1e−05
2e−05

D

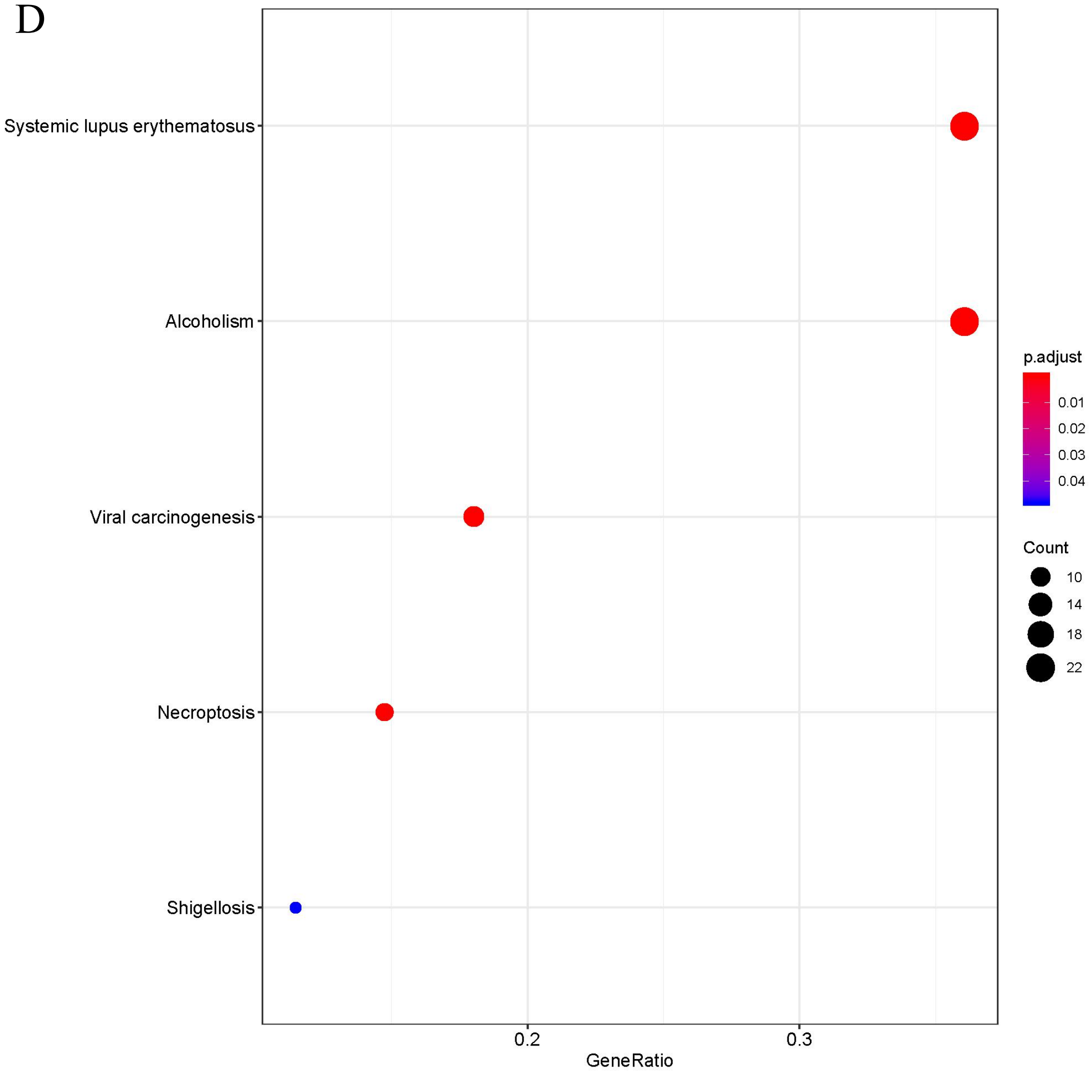

Supplementary Figure S1. Functional analysis of miRNAs and mRNAs regulated by Enh_{p53}. **(A)** GO analysis of miRNAs regulated by Enh_{p53}. **(B)** KEGG analysis of miRNAs regulated by Enh_{p53}. **(C)** GO analysis of mRNAs regulated by Enh_{p53}. **(D)** KEGG analysis of mRNAs regulated by Enh_{p53}.
